# Regenerating the epidural space: Promise and perspective on amniotic fluid for low back pain^☆^

**DOI:** 10.1016/j.inpm.2025.100627

**Published:** 2025-08-08

**Authors:** Alexandre J. Bourcier, Zbigniew M. Kirkor

**Affiliations:** a Department of Medicine, University of Pittsburgh Medical Center, Pittsburgh, PA, USA; b Kirkor Consulting LTD, London, UK

Dear Editor,

Amniotic fluid and membrane-derived products have gained increasing attention in regenerative medicine for their anti-inflammatory, anti-scarring, and potential tissue-repair properties. In this context, the recent study by Buttermann et al. offers an important exploratory look at epidural amniotic fluid injections for chronic low back pain [1]. The authors report meaningful improvements in pain and function following fluoroscopically guided injections using a commercially available amniotic fluid preparation.

While still early-stage, this study opens the door for broader consideration of biologic therapies beyond platelet-rich plasma (PRP) and mesenchymal stromal cells (MSCs) [2]. The appeal of amniotic fluid lies in its immune-privileged nature and bioactive matrix, potentially allowing for anti-inflammatory modulation without the regulatory hurdles of cell-based therapies. However, as with all regenerative products, enthusiasm must be balanced with a rigorous understanding of mechanism, clinical context, and reproducibility.

A key strength of this study is the use of real-world data from a spine-focused practice. The authors provide outcomes in patients with recalcitrant axial low back pain, a notoriously difficult population. However, the absence of a control group makes it difficult to separate therapeutic effect from regression to the mean, placebo, or the natural course of symptoms. Furthermore, epidural delivery introduces additional complexity, it remains unclear how much of the observed benefit is attributable to the injectate's bioactive composition versus nonspecific effects such as the dilution or washout of inflammatory mediators.

Another issue is comparative effectiveness. With PRP increasingly studied in discogenic pain and MSCs showing early promise in degenerative disc disease, how does amniotic fluid stack up? Head-to-head comparisons remain rare, and current studies often vary in cell concentration, product source, and delivery method [3]. As regenerative spine interventions move forward, standardization of protocols and outcome reporting will be essential for meaningful interpretation.

It's also worth noting the regulatory gray zone in which many amniotic products exist [4,5]. While these injectables are marketed as minimally manipulated allografts, their clinical claims often outpace the level of evidence. This highlights the need for post-marketing surveillance and independent registry data to track both efficacy and safety over time.

Ultimately, Buttermann et al. deserve credit for advancing a conversation that blends biological innovation with procedural pain management. Future research should aim for randomized, placebo-controlled trials with standardized endpoints and longer follow-up. Until then, regenerative physicians should remain cautiously optimistic, maintaining a balance between innovation and evidence.

## Declaration of generative AI and AI-assisted technologies in the writing process

During the preparation of this work the authors used ChatGPT in order to improve writing style and proofread for any inconsistencies. After using ChatGPT, the authors reviewed and edited the content as needed and take full responsibility for the content of the publication.

## Funding

This research did not receive any specific grant from funding agencies in the public, commercial, or not-for-profit sectors.

## Declaration of competing interest

The authors declare that they have no known competing financial interests or personal relationships that could have appeared to influence the work reported in this paper.
